# Foregut duplication of the stomach diagnosed by endoscopic ultrasound guided fine-needle aspiration cytology: case report and literature review

**DOI:** 10.1186/1477-7819-11-33

**Published:** 2013-02-02

**Authors:** Vincenzo Napolitano, Angelo M Pezzullo, Pio Zeppa, Pietro Schettino, Maria D’Armiento, Antonietta Palazzo, Cristina Della Pietra, Salvatore Napolitano, Giovanni Conzo

**Affiliations:** 1 Department of General and Specialistic Surgery, School of Medicine, Second University of Naples, 5 S. Pansini Street, 80100, Naples, Italy; 2 Department of Pathology, School of Medicine, Federico II University of Naples, 80100, Naples, Italy

**Keywords:** Gastric duplication cyst, Foregut duplication cysts, Pseudostratified columnar ciliated epithelium, Laparoscopic surgery, Endoscopic ultrasound-guided fine-needle aspiration cytology

## Abstract

Gastric duplication cyst (GDC) with a pseudostratified columnar ciliated epithelium is an uncommon malformation supposed to originate from a respiratory diverticulum arising from the ventral foregut. Morphologic appearance of GDCs is variable, depending on the density of their contents. GDCs are often misdiagnosed as solid masses by imaging techniques, and as a consequence they may be wrongly overtreated. We report our case of a 56-year-old man with a 5 cm hypoechoic mass of the gastroesophageal junction, incidentally detected by transabdominal ultrasonography. Neither transabdominal ultrasonography nor magnetic resonance clearly outlined the features of the lesion. The patient underwent endoscopic ultrasound (EUS), which showed a hypoechoic mass arising from the fourth layer of the anterior gastric wall, just below the gastroesophageal junction. According to EUS features, a diagnosis of gastrointestinal stromal tumor was suggested. EUS-guided fine-needle aspiration cytology revealed a diagnosis of GDC with pseudostratified columnar ciliated epithelium. We therefore performed an endoscopically-assisted laparoscopic excision of the cyst.

In conclusion, whenever a subepithelial gastric mass is found in the upper part of the gastric wall, a duplication cyst, although rare, should be considered. In this case, EUS-guided fine-needle aspiration cytology could provide a cytological diagnosis useful to arrange in advance the more adequate surgical treatment.

## Background

Duplications of the alimentary tract are relatively rare congenital anomalies. Those located in the stomach are very uncommon, constituting between 4 and 9% of all intestinal duplications [1]. The structure of a gastric duplication cyst (GDC) consists of a smooth muscle coat lined by a mucous membrane, in most cases containing a typical gastric epithelium [2], although a small intestinal or colonic mucosa may also be present. Generally, they are single and do not communicate with gastric lumen. Exceptionally, in GDCs a pseudostratified columnar ciliated epithelium, more commonly present in the esophageal duplication cysts, can be found. According to Cunningham and colleagues [3], GDCs lined by pseudostratified columnar ciliated epithelium could be better defined as foregut duplication cysts (FDCs) of the stomach.

Diagnosis of a gastric duplication may be difficult even using the most advanced imaging techniques, including endoscopic ultrasound (EUS) [4-6]. Here we report a case of GDC with respiratory epithelium, misdiagnosed as a gastrointestinal stromal tumor (GIST) at EUS. The EUS-guided fine-needle aspiration subsequently performed led to a definite preoperative diagnosis, allowing a proper conservative endoscopically-assisted laparoscopic resection of the cyst.

## Case presentation

A 56-year-old man, with a history of B-related chronic hepatitis under antiretroviral treatment, was referred to our surgical department by the Infectious Diseases Unit, where he was under follow-up. The patient did not complain of any symptom concerning the gastrointestinal tract. During an abdominal ultrasonography, a hypoechoic round-shaped mass 4.7 cm in size, with regular margins, located between the left lobe of the liver and the anterior surface of the pancreatic body, was found. Magnetic resonance imaging confirmed the presence of a cystic mass with complex content, located anteriorly to the gastroesophageal junction.

**Figure 1:**
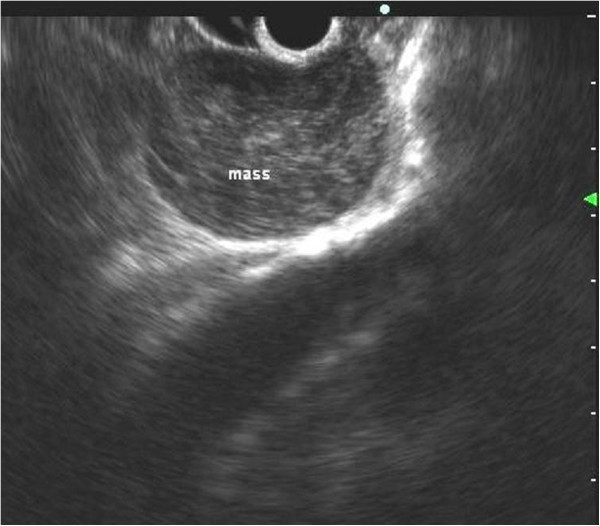
**Endoscopic ultrasound features.** A hypoechoic mass with a slight heterogeneous texture developing within the gastric wall.

**Figure 2:**
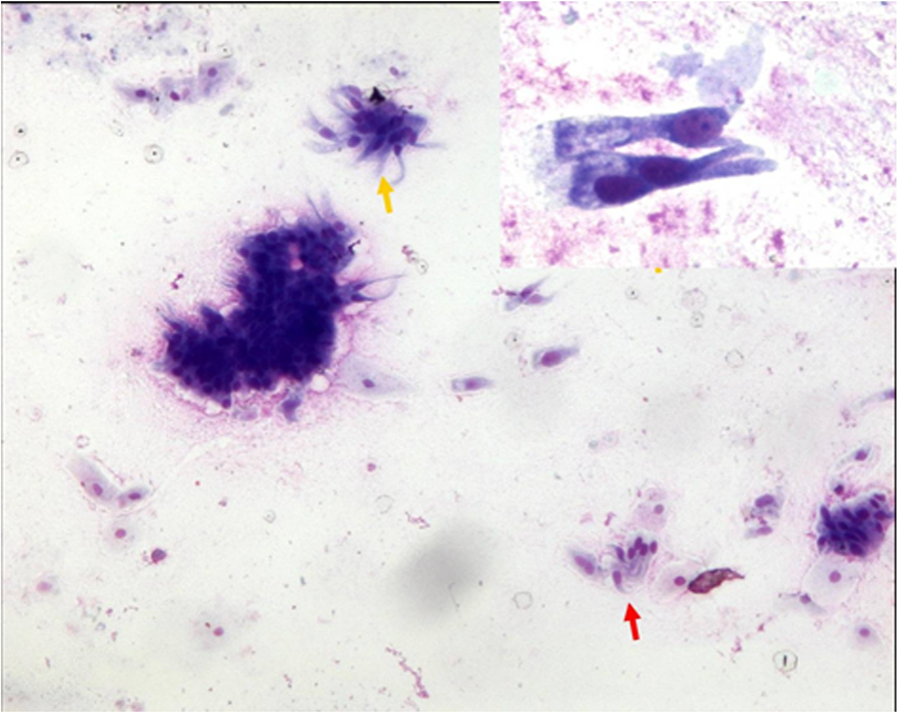
**Cytology on fine-needle aspiration sampling.** Isolated and aggregated cylindrical ciliated cells (yellow arrows) in a background containing debris and squamous cells. Note characteristic palisade arrangement (red arrow) (Diff.Quik stain, × 270). Inset: Cylindrical cells show long cilia and brush borders similar to the ciliated cells of the respiratory tract (Diff.Quik stain, × 430).

The patient was then submitted to EUS, in order to better define the structure of the lesion and its relationship with the adjacent organs. EUS showed a hypoechoic mass with slightly heterogeneous internal echoes and regular margins, located just below the gastroesophageal junction (Figure 1). The lesion measured about 4.5 cm and seemed to be contiguous to the fourth layer of the gastric wall (muscolaris propria). On the basis of the morphologic evaluation, a diagnosis of GIST was suggested. As is usual in a case of suspected GIST involving the upper part of the gastric wall, we tried to obtain diagnostic confirmation through EUS-guided fine-needle aspiration cytology (FNAC). The puncture was practiced using a 22 G needle, which, unexpectedly, penetrated very easily into the mass as it was cystic. We were able to aspirate only a few milliliters of a clear mucus-like fluid, and then a cytological sampling was made from the cystic wall. The collected material was judged adequate by an onsite cytopathologist. The cytological smear showed cylindrical cells isolated or aggregated in small groups with a palisade organization (Figure 2). These cells showed long cilia and brush borders similar to the ciliated cells of the respiratory tract. These basal cells had oval nuclei with finely dispersed chromatin and small nucleoli, if any. The background consisted of proteinaceous material containing debris, crystal formations and engulfed hystiocytes. On the basis of these features, a diagnosis of duplication cyst with respiratory epithelium was made.

Later on, the patient underwent a surgical intervention carried out by an open laparoscopic approach with a transhumbelical Hasson trocar (without a Verres needle to obtain the pneumoperitoneum) and four additional trocars (two of 10 mm and two of 5 mm). Once the lesion was clearly identified, the overlying serosa was cut by a harmonic scalpel (Harmonic Ace; Ethicon Endo-Surgery, Cincinnati, OH, USA). Through a cautious dissection performed under endoscopic control in order to keep the cyst intact, to prevent perforation of the gastric wall, the mass was totally exposed and then completely resected using a linear endoscopic stapler (Echelon™ 60; Ethicon Endo-Surgery). The surgical procedure was completed by performing a Dor fundoplication. The patient had an uneventful postoperative recovery and was discharged on the seventh postoperative day.

**Figure 3:**
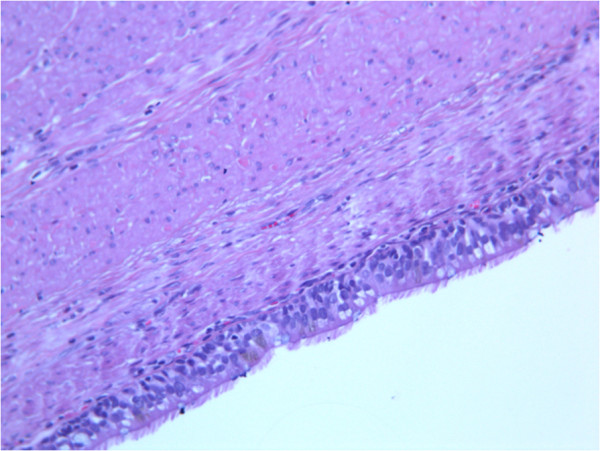
**Histology on surgical specimen.** Histological sections of the cystic wall showing a cylindrical pseudostratified mucosa on a muscular wall. Epithelial cells show cilia as in the ontogenesis of primitive gut (hematoxylin and eosin, × 270).

Pathologic examination of the surgical specimen revealed macroscopically a cystic lesion 5 cm × 3 cm × 3 cm in size with a mucoid content. Microscopically the cystic wall consisted of mucosa, subepithelial connective tissue, a layer of smooth muscle and an outer fibrous capsule. Focally the mucosa was lined by gastric foveolar epithelium with cardial glands but most of the cystic wall was lined by a pseudostratified columnar ciliated epithelium (Figure 3). These features were consistent with a diagnosis of foregut duplication cyst of the stomach.

## Discussion

GDCs with a pseudostratified columnar ciliated epithelium (also named foregut duplication cysts of the stomach) are supposed to originate from a respiratory diverticulum, arising from the ventral foregut [7]. This type of gastric duplication is very rare. Including the present report, there have so far been only 21 reported cases. Evaluating patient data, summarized in Table 1, gastric FDCs seem to be a late-onset disease with no differences in relation to gender. In most cases these lesions are located in the upper part of the stomach: at the level of the cardia, near the gastroesophageal junction, or in the anterior or posterior wall of the fundus. Very often, as in our patient, they are asymptomatic and incidentally found. Symptoms, when present, are not specific, including mainly abdominal or epigastric pain. Consistency of FDCs can range from a thin free-flowing fluid to thick proteinaceous material [8].

**Table 1:** Gastric duplication cyst lined by pseudostratified columnar ciliated epithelium

**References**	**Age (year)**	**Gender**	**Complaint**	**Location**
Gensler *et al.*, 1966 [8]	46	F	No	NGEJ, GC
Takahara *et al.*, 1996 [9]	25	M	No	Fundus, PW
Kim *et al.*, 2000 [10]	35	M	Epigastric pain	NGEJ, LC
Hedayati *et al.*, 2003 [11]	59	F	No	Fundus, LC
Melo *et al.*, 2005 [12]	39	F	No	Fundus
Rubio *et al.*, 2005 [13]	26	M	Epigastric pain	NA
Song *et al.*, 2005 [14]	62	F	No	NGEJ, LC
Lee *et al.*, 2006 [15]	38	F	No	Cardia, LC
Cunningham *et al.*, 2006 [3]	63	F	Fever, abdominal pain	Fundus, PW
Wakabayashi *et al.*,2007 [16]	37	M	Epigastric pain	NGEJ, LC
Hall *et al.*, 2007 [17]	40	M	Epigastric discomfort	NGEJ, LC
Theodosopoulos *et al.*,2007 [18]	46	F	Vomiting	(1) Fundus PW;
				(2) Gastrosplenic lig.
Sato *et al.*, 2008 [19]	60	F	No	Cardia, LC
Murakami *et al.*, 2008 [20]	72	F	No	Middle body, LC
Shibahara *et al.*, 2009 [21]	43	M	Epigastric pain	Cardia, LC
Mardi *et al.*, 2010 [22]	42	M	Left lumbar pain	Cardia, LC
Jiang *et al.*, 2010 [23]	25	F	Epigastric pain	Fundus
Jiang et al. 2011 [24]	76	M	No	NGEJ, LC
Khoury et al. 2011 [7]	29	M	Abd pain	Fundus GC
	26	F	Epigastric pain	Middle body LC
Present	56	M	No	NGEJ, AW

M: male; F: female;; NGEJ: near gastroesophageal junction; LC: lesser curvature; GC: greater curvature; PW: posterior wall; AW : anterior wall, NA : not available.

Despite advances in imaging, cysts that contain solid secretions can often be misclassified as soft tissue masses. A rate of computed tomography misdiagnosis ranging from 43 to 70% of cases has been reported [9,10]. Magnetic resonance imaging does not seem to significantly improve diagnostic accuracy [6]. Therefore, in the majority of the reported cases, a definite diagnosis was made only during surgical resection or by pathological examination on surgical specimens [11-14]. EUS is currently the best available method for the diagnosis of the subepithelial lesions of the gastrointestinal tract. This technique has also been proved to be superior to computed tomography scan in distinguishing cystic from solid masses [1], but the diagnostic accuracy of EUS is affected by the variation of intracystic contents. The use of contrast-enhanced EUS may also be very useful in the differential diagnosis of digestive diseases [15,16]. However, we did not find in the literature any paper discussing the role of contrast-enhanced EUS in the diagnostic evaluation of GDCs.

On the basis of EUS morphologic findings alone, a GDC may be misdiagnosed as a GIST, which is the most common gastric subepithelial lesion, as in the case reported by Jiang and colleagues [17]. In the present case, EUS findings also suggested a diagnosis of GIST. Since the surgical treatment of GISTs involving the upper part of the gastric wall may require an extended gastric resection, we performed EUS-guided FNAC in order to confirm the diagnosis. While inserting the needle we realized that the presumed GIST was a cystic lesion, and the cytological sampling led to a diagnosis of GDC with respiratory epithelium. There are only few case reports concerning EUS-guided FNAC of gastrointestinal duplication cysts [1,10,18-20], but since respiratory-type cells or detached ciliary tufts are visualized in cytologic preparations, a definite diagnosis can be easily made [10]. Pitfalls can occur if the cyst is lined by gastric epithelium only, as in the case described by Wang and colleagues [1]. EUS-guided FNAC led to a cytological misdiagnosis of gastric mucinous neoplasm.

Owing to the report of gastric cancer arising in gastric duplication [2,21-23], surgery is nowadays considered the standard treatment for these lesions [24]. The possibility of a malignant transformation is related to the presence of a gastric-type lining epithelium. Ponder and Collins therefore suggested that surgery is not necessary if respiratory epithelium is recognized on EUS-guided FNAC [20]. Nevertheless, it has been shown in the FDCs of the stomach that pseudostratified columnar ciliated epithelium may be associated with gastric epithelium [24], which could be missed by cytological sampling. For this reason, it may be that a complete surgical excision of the cyst should be recommended; also, in selected cases, some authors consider its observation as a reasonable option. A surgical procedure that does not require a gastric resection can be easily undertaken by a laparoscopic approach, as performed in this case.

## Conclusion

In summary, GDCs – particularly those with respiratory epithelium – represent a rare disease, often misdiagnosed as GISTs, which are more common. Nevertheless, these lesions should be considered in the differential diagnosis of subepithelial gastric masses, especially if located in the upper part of the gastric wall. Using this technique we will not take any chance on treating a FDC of the stomach through unnecessary extended gastric resection.

## Consent

Written informed consent was obtained from the patient for publication of this case report and any accompanying images. A copy of the written consent is available for review by the Editor-in-Chief of this journal.

## Abbreviations

EUS: endoscopic ultrasound; FDC: foregut duplication cyst; FNAC: fine-needle aspiration cytology; GDC: gastric duplication cyst; GIST: gastrointestinal stromal tumor.

## Competing interests

The authors declare that they have no competing interests.
